# Successful Management of Chronic Wounds by an Autophagy-Activating Magnetized Water-Based Gel in Elderly Patients: A Case Series

**DOI:** 10.7759/cureus.55937

**Published:** 2024-03-11

**Authors:** Piercarlo Minoretti, Manuel Gómez Serrano, Andrés Santiago Sáez, Miryam Liaño Riera, Ángel García Martín

**Affiliations:** 1 Occupational Health, Studio Minoretti, Oggiono, ITA; 2 Legal Medicine, Psychiatry, and Pathology, Complutense University of Madrid, Madrid, ESP; 3 Legal Medicine, Hospital Clinico San Carlos, Madrid, ESP

**Keywords:** trauma-related injuries, venous ulcers, pressure ulcers, magnetized water, autophagy, elderly, wound healing

## Abstract

Chronic wounds pose a significant threat to human health, particularly for the elderly, and require extensive healthcare resources globally. Autophagy, a key molecular player in wound healing, not only offers a defense against infections but also contributes to the deposition of the extracellular matrix during the proliferative phase. Additionally, it promotes the proliferation and differentiation of endothelial cells, fibroblasts, and keratinocytes. We have recently shown that applying magnetized saline water topically can trigger autophagy in intact skin. In this case series, we document the successful management of five non-infected, difficult-to-heal wounds in elderly patients using a topical autophagy-stimulating gel containing 95% magnetized saline water. The treated wounds included pressure ulcers, venous ulcers, and trauma-related injuries that had shown minimal or no improvement with standard wound therapies over a prolonged period. Application of the autophagy-stimulating gel promoted wound healing, as indicated by reduced fibrous and necrotic tissue, granulation tissue formation, re-epithelialization, and partial or complete wound closure. These preliminary case studies suggest that a topical gel containing magnetized saline water, which promotes autophagy, may aid healing of chronic wounds in elderly patients. Further investigation is warranted to explore the potential of this novel approach, as it may offer a valuable addition to the existing arsenal of wound care treatments for the aging population, particularly in addressing difficult-to-heal wounds.

## Introduction

Chronic wounds, defined as those that fail to proceed through an orderly and timely process to produce anatomic and functional integrity, are characterized by prolonged or excessive inflammation and reduced cellular responses to healing stimuli from dermal and/or epidermal tissues [1, 2]. Estimates indicate that 1−2% of individuals will suffer from a chronic wound at some point during their lifetime [3]. Moreover, the expanding population of adults aged 65 years and older has positioned impaired wound healing as a major global health issue [4]. Beyond posing risks of severe complications such as infection, gangrene, hemorrhage, and potential amputation, chronic wounds levy a substantial burden on healthcare systems [5]. The management of difficult-to-heal wounds frequently necessitates sophisticated therapeutic modalities including growth factors, extracellular matrices, bioengineered skin substitutes, and negative pressure therapy [6, 7]. While these advanced treatments carry potential benefits, their high costs and limited accessibility in low-resource environments pose additional challenges.

Autophagy, a highly conserved cellular recycling process, has a complex and multifaceted role across the wound healing process [8]. In the initial inflammatory stage, it functions as an innate defense mechanism against pathogens that could impede healing while also tempering inflammation to prevent excessive tissue damage [9]. As healing transitions into the proliferative phase, autophagy facilitates extracellular matrix deposition and promotes the proliferation and differentiation of endothelial cells, fibroblasts, and keratinocytes [9]. Through these mechanisms, autophagy is pivotal for re-epithelialization and the ultimate restoration of skin barrier integrity [10].

Recently, we have shown that the topical application of magnetized saline water can stimulate autophagy in intact skin [11]. Starting from the assumption that autophagy may facilitate multiple aspects of wound healing [8-10], we hypothesized that activating this process via a topical gel containing 95% magnetized saline water could aid in healing difficult-to-treat wounds where autophagy is frequently impaired. In this case series, we document the successful management of five non-infected, difficult-to-heal wounds in elderly patients using a topical autophagy-stimulating gel containing 95% magnetized saline water. The treated lesions included pressure ulcers, venous ulcers, and trauma-related injuries that had shown minimal or no improvement with standard wound therapies over a prolonged period. Application of the autophagy-stimulating gel promoted wound healing, as indicated by reduced necrotic tissue, granulation tissue formation, and partial or complete wound closure. Our data provide proof-of-concept evidence that activating autophagy may promote wound healing in humans.

## Case presentation

Investigational product

The investigational product was a topical gel (Aquavis, Brescia, Italy) containing 95% magnetized saline water. We have previously shown that topical application of magnetized saline water to intact skin can induce autophagy, as evidenced by an increased expression of the autophagy markers Beclin-1 and LC3B [11]. All participants had their wounds cleaned with distilled water before applying a sufficient quantity of the gel to the wound area twice daily for three months or until partial or complete wound healing occurred, whichever was earlier. After the application of the product, the treated area was dressed with sterile gauze per standard wound care protocols. A summary of the five reported cases is provided in Table 1.

**Table 1:** General characteristics of the five reported patients.

	Case 1	Case 2	Case 3	Case 4	Case 5
Age, years	82	88	85	87	67
Sex	Woman	Woman	Woman	Man	Woman
Socioeconomic level	Primary school	Secondary school	Secondary school	Primary school	Secondary school
Household size	4	3	4	4	Long-term care facility
Coexistence with animals	None	None	None	None	None
Comorbidities	Left-sided hemiplegia, chronic heart failure	Obesity, Parkinson’s disease, chronic venous insufficiency	Osteoarthritis, poorly controlled hypertension, chronic venous insufficiency	Chronic obstructive pulmonary disease, vascular dementia, chronic ischemic heart disease	Post-cardiac arrest brain injury
Time with chronic injury	Ten months	Twelve months	Five months	Twelve months	Eleven months
Treatment frequency	Twice daily	Twice daily	Twice daily	Twice daily	Twice daily
Treatment time	Four months	Seven months	One month	Seven months	Six months

Case report 1

**Figure 1:**
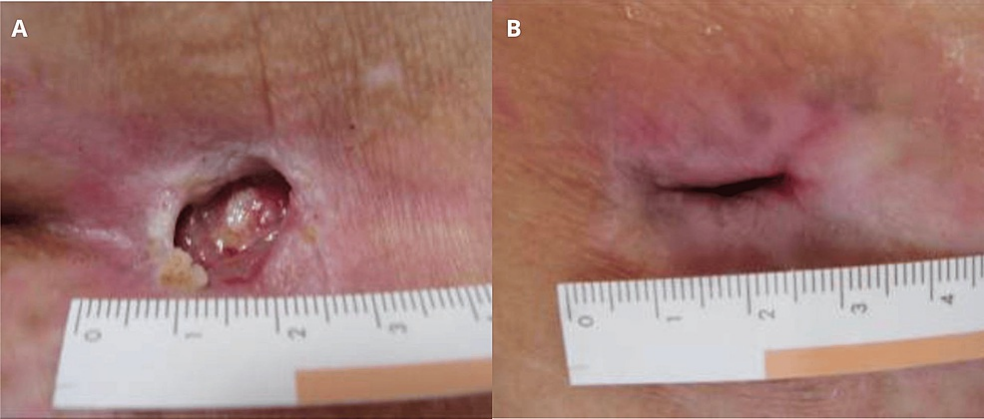
Sacral pressure ulcer (case 1). Stage 4 sacral pressure ulcer at baseline (A). Significant reduction of the ulcer size after application of the gel over four months (B).

An 82-year-old woman with a history of left-sided hemiplegia and chronic heart failure developed a stage 4 sacral pressure ulcer after a prolonged period of immobility (Figure 1A). Conventional treatments including debridement and silver ion dressings yielded limited results. After the application of the gel over four months, the ulcer size was significantly reduced (Figure 1B).

Case report 2

**Figure 2:**
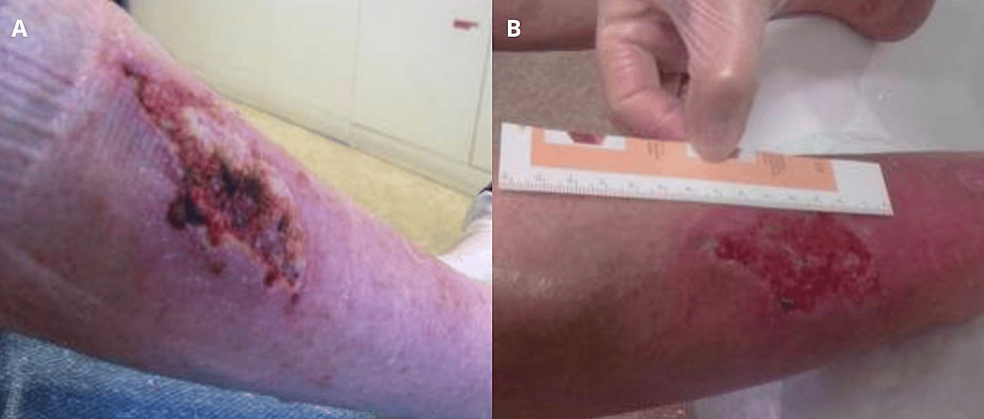
Unintentional lower limb injury (case 2). Baseline aspect of the lesion (A). Development of granulation tissue across the affected area after application of the gel over seven months (B).

An 88-year-old woman affected by obesity, Parkinson’s disease, and chronic venous insufficiency sustained an unintentional lower limb injury (Figure 2A). Following debridement, the lesion underwent routine care and compression therapy. Due to limited response, application of the gel to the wound site was attempted. Over the course of seven months, this treatment led to notable progress, as evidenced by the development of granulation tissue across the affected area (Figure 2B).

Case report 3

**Figure 3:**
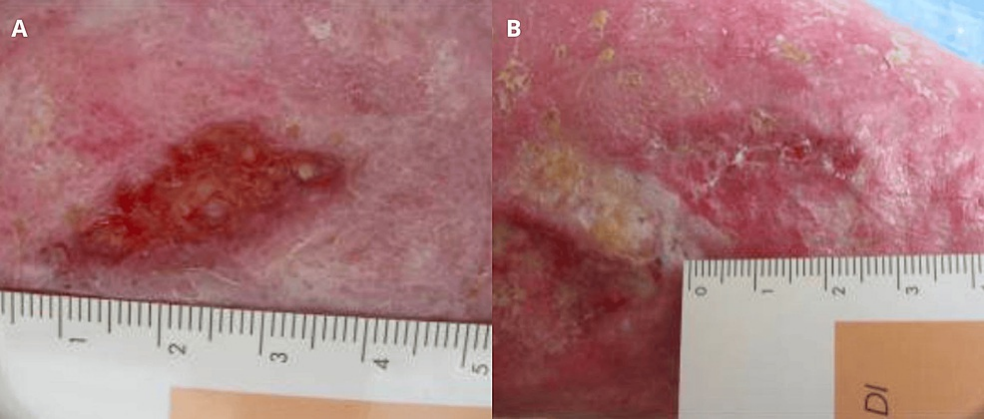
Venous leg ulcer (case 3). Baseline aspect of the lesion (A). Improvement of skin integrity after application of the gel for one month (B).

An 85-year-old woman with a history of osteoarthritis, poorly controlled hypertension, and chronic venous insufficiency in her lower limbs was severely impaired in her activities of daily living and required constant assistance from a caregiver. She developed post-phlebitic syndrome, resulting in multiple venous ulcers on her left lower limb (Figure 3A). After one month of gel application, the skin integrity of her limb appeared to improve (Figure 3B).

Case report 4

**Figure 4:**
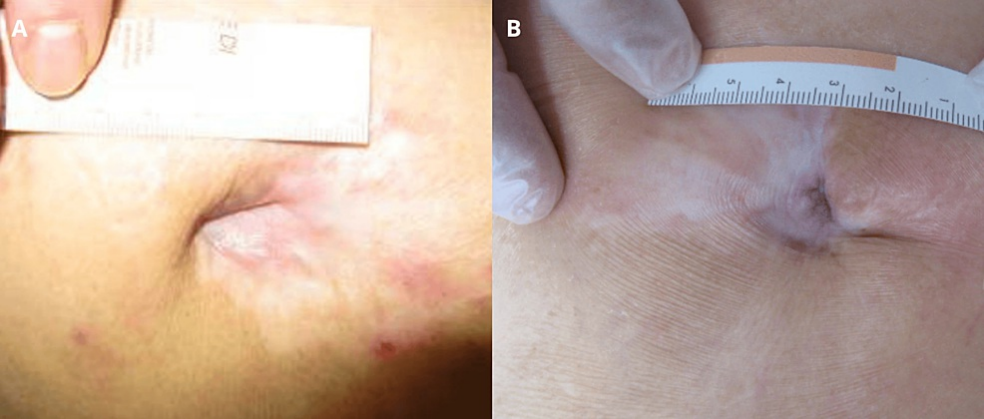
Trochanteric pressure ulcer (case 4). Baseline aspect of the lesion (A). Signs of healing and reduction in size after application of the gel over seven months (B).

A bedridden 87-year-old man with chronic obstructive pulmonary disease, vascular dementia, and chronic ischemic heart disease developed a trochanteric pressure ulcer (Figure 4A). Following seven months of standard wound care including gel application, the ulcer demonstrated signs of healing and reduction in size (Figure 4A).

Case report 5

**Figure 5:**
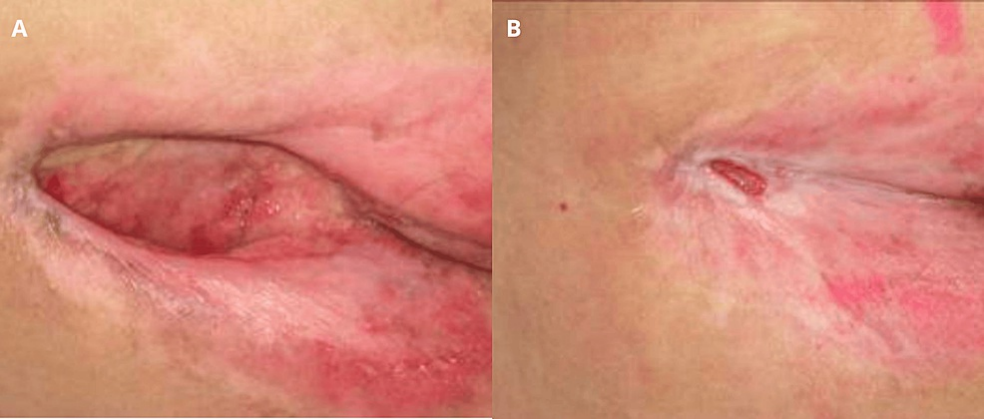
Sacral pressure ulcer (case 5). Stage 4 sacral pressure ulcer at baseline (A). Significant reduction of the ulcer size after application of the gel over six months (B).

A 67-year-old woman suffering from post-cardiac arrest brain injury developed a large stage 4 sacral pressure ulcer (Figure 5A). Due to limited response to standard treatment, the gel was applied as an attempted alternative therapy. After six months, the pressure ulcer demonstrated notable improvement (Figure 5B).

## Discussion

Wound healing is an intricate process encompassing tissue protection, regeneration, and reorganization, culminating in the restoration of tissue integrity [7, 12]. Any alteration in these stages can lead to incomplete healing, manifesting as chronic ulcers [6], or excessive healing, resulting in hypertrophic scarring [13]. Sylakowski and Wells have recently introduced a conceptual framework emphasizing the pivotal role of matrix-autophagy modulation in both physiological and pathological wound healing [14]. They postulate that matrix-mediated cellular macro-autophagy is crucial during the tissue replacement and resolution phases of healing, influencing cellular activities and programmed cell death [14]. Interestingly, a variety of biomaterials have demonstrated the potential to expedite the healing process by promoting autophagy [15]. These materials include but are not limited to, nonfouling zwitterionic sulfated poly(sulfobetaine methacrylate) hydrogels, thermo-sensitive chitosan hydrogels embedding with poly lactic-co-glycolic acid microspheres, metal-organic frameworks, and silver nanoparticles [15]. However, despite the promising findings from in vitro studies, animal models, and observations in human tissues [8-10], there is a scarcity of direct clinical evidence supporting the effectiveness of autophagy activation as a therapeutic strategy for enhancing wound healing in human patients.

Considering the potential of magnetized saline water as a straightforward approach to induce autophagy via topical application on intact skin [11], this case series was designed to evaluate, for the first time, the effectiveness of its gel formulation in enhancing wound healing. In the five elderly patients described in this study, each presenting with wounds that had not responded to conventional treatments, the application of the magnetized water gel yielded encouraging healing outcomes, demonstrating its promising efficacy in wound management. These proof-of-concept findings are in line with the theory proposed by Mijaljica et al., which identifies autophagy as a critical mechanism in transitioning chronic wounds into an acute healing phase [9]. In addition to activating autophagy, it is also possible that the magnetized water gel may enhance moisture retention, thereby fostering an ideal environment for cell proliferation, migration, and extracellular matrix reorganization, all while simultaneously mitigating inflammation. Such conditions are vital for effective and swift wound healing, highlighting the critical role of maintaining optimal moisture levels within wounds.

However, it is crucial to acknowledge that our results are based on a series of case studies, rather than a controlled clinical trial. These observations, emerging from routine clinical practice, emphasize the need for additional research. Given these preliminary results, we advocate for the conduct of rigorous clinical trials to definitively ascertain the therapeutic potential of autophagy-activating gels in the treatment of challenging wounds.

## Conclusions

These preliminary case studies suggest that a topical gel containing magnetized saline water, which promotes autophagy, may aid healing of chronic wounds in elderly patients. Further investigation is warranted to explore the potential of this novel therapeutic approach, as it may offer a valuable addition to the current armamentarium of treatments for the aging population, particularly in addressing refractory injuries.
